# Exosome Uptake Depends on ERK1/2-Heat Shock Protein 27 Signaling and Lipid Raft-mediated Endocytosis Negatively Regulated by Caveolin-1*[Fn FN2]

**DOI:** 10.1074/jbc.M112.445403

**Published:** 2013-05-07

**Authors:** Katrin J. Svensson, Helena C. Christianson, Anders Wittrup, Erika Bourseau-Guilmain, Eva Lindqvist, Lena M. Svensson, Matthias Mörgelin, Mattias Belting

**Affiliations:** From the Departments of ‡ Clinical Sciences, Section of Oncology,; ¶ Experimental Medical Science, Section of Immunology, and; ‖ Clinical Sciences, Section of Clinical and Experimental Infectious Medicine, Lund University, SE-22184 Lund, Sweden,; the § Program in Molecular and Cellular Medicine, Boston Children's Hospital, Harvard Medical School, Boston, Massachusetts 02115, and; the ** Department of Oncology, Skåne University Hospital, SE-22241 Lund, Sweden

**Keywords:** Caveolin, Endocytosis, Exosomes, Glioblastoma, Vesicles, Microvesicles

## Abstract

The role of exosomes in cancer can be inferred from the observation that they transfer tumor cell derived genetic material and signaling proteins, resulting in *e.g.* increased tumor angiogenesis and metastasis. However, the membrane transport mechanisms and the signaling events involved in the uptake of these virus-like particles remain ill-defined. We now report that internalization of exosomes derived from glioblastoma (GBM) cells involves nonclassical, lipid raft-dependent endocytosis. Importantly, we show that the lipid raft-associated protein caveolin-1 (CAV1), in analogy with its previously described role in virus uptake, negatively regulates the uptake of exosomes. We find that exosomes induce the phosphorylation of several downstream targets known to associate with lipid rafts as signaling and sorting platforms, such as extracellular signal-regulated kinase-1/2 (ERK1/2) and heat shock protein 27 (HSP27). Interestingly, exosome uptake appears dependent on unperturbed ERK1/2-HSP27 signaling, and ERK1/2 phosphorylation is under negative influence by CAV1 during internalization of exosomes. These findings significantly advance our general understanding of exosome-mediated uptake and offer potential strategies for how this pathway may be targeted through modulation of CAV1 expression and ERK1/2 signaling.

## Teaser

**Background:** Exosome vesicles can transfer molecular information previously shown to stimulate tumor development; however, the mechanism of exosome uptake is unknown.

**Results:** Mammalian cells internalize exosomes through lipid raft-mediated endocytosis negatively regulated by caveolin-1.

**Conclusion:** Our findings provide novel insights into cellular uptake of exosomes.

**Significance:** Our data provide potential strategies for how the exosome uptake pathway may be targeted.

## Introduction

The process of endocytosis involves multiple mechanisms in mammalian cells differing in the type of cargo and fate of cargo upon internalization. Described mechanisms of classical endocytosis include clathrin-dependent endocytosis, macropinocytosis, clathrin-independent endocytosis pathways such as caveolae-mediated uptake associated with lipid rafts (or cholesterol-enriched membrane microdomains) in the plasma membrane and non-classical pathways involving non-clathrin, non-caveolae-mediated endocytosis (1–4). Secreted vesicles, here classified as exosomes (also referred to as microvesicles, shedded vesicles, ectosomes, microparticles, plasma membrane-derived vesicles) are complex biological vehicles enclosed with cytoplasmic components and genetic material (5–8). The putative function of exosomes in cancer are based on the recently described findings of the transfer of genetic material and signaling proteins, resulting in *e.g.* increased angiogenesis and metastasis (8–12). Exosomes are released from multivesicular bodies (MVBs)^2^ upon their fusion with the plasma membrane (13). Recent studies suggest that released vesicles may transfer functional RNA as well as transmembrane proteins contributing to the propagation of a transformed cell phenotype (9, 10, 14). Accordingly, exosomes can be regarded as multi-purpose delivery vehicles in analogy with an endogenous virus-like particle infecting cells in the surrounding environment. Importantly, recent reports have documented key processes involved in exosome release and have identified similarities in topology and mechanisms to retroviral budding (15, 16). It is still controversial whether vesicular uptake is cell type specific (17), and whether it involves membrane fusion or endocytosis (18, 19). Thus, studies elucidating the mechanisms involved in exosome uptake remain an important challenge. We have previously reported on microvesicle-induced pro-angiogenic signaling; a process highly relevant in the most aggressive brain tumor type GBM (20). Here we set out to elucidate the unknown mechanisms of the uptake of GBM cell-derived vesicles and the signaling events involved in the internalization process.

## EXPERIMENTAL PROCEDURES

### 

#### 

##### Cell Culture

Human umbilical vein endothelial cells (HUVECs, Lonza) were cultured in endothelial basal medium supplemented with 10% heat-inactivated fetal bovine serum (FBS), 2 mm l-glutamine, 100 units/ml penicillin, 100 μg/ml streptomycin, 10 ng/ml hydrocortisone, and 20 μg/ml human recombinant EGF. Human cervix adenocarcinoma (HeLa, ATCC), HeLa cells previously generated to stably overexpress CAV1-YFP (21), human GBM cells (U87 MG, ATCC) and wild-type (WT) or CAV1 knock out (cav-1 (−/−)) mouse embryonic fibroblasts (MEF, ATCC) were cultured in DMEM supplemented with 10% FBS, 2 mm l-glutamine, 100 units/ml penicillin, 100 μg/ml streptomycin (growth medium). CHO-K1 and COS-7 cells were cultured in F12K supplemented with 10% FBS, 2 mm l-glutamine, 100 units/ml penicillin, 100 μg/ml streptomycin. U87 MG shRNA negative control (NC) and shRNA CAV1 were routinely cultured in growth medium supplemented with 1 μg/ml puromycin. U87 MG cells transfected with a CD63-mCherry plasmid were sorted with FACSAria by fluorescence intensity and clones were further selected by neomycin resistance. U87 MG-CD63-mCherry cells were routine cultured in growth medium supplemented with 1000 μg/ml G418. All cells were cultured in a humidified incubator with 5% CO_2_ at 37 °C.

##### Antibodies and Reagents

Antibodies for CAV1 (ab2910), CD63 (ab8219), TSG101 (ab30871), clathrin heavy chain (ab21679), p-HSP27 (ab17937), HSP27 (ab5579), flotillin-1 (ab41927), calnexin (ab2798), β-actin (ab8227), and α-tubulin (ab7291) were from Abcam. TF antibody (10H10) and siRNA for clathrin (sc-35067) were from Santa Cruz Biotechnology. Secondary antibodies conjugated with 5 or 15 nm gold were from Electron Microscopy Sciences, Fort Washington, PA. Total ERK1/2 antibody (9102) and siRNA for HSP27 (#6526S) were from Cell Signaling. Normal mouse IgG was from Molecular Probes. siRNA for CD63 (#4392420, ID:s2701), Mission Lentiviral transduction particles (SHCLNV) (shRNA) for CAV1 (NM_001753 clone TRCN000008002) and negative control (NC) (SHCO16V, pLKO.1-puro), phosphorylated (Thr183/Tyr185) ERK1/2 antibody (M8159), PKH67 Green Fluorescent cell linker midi kit, PKH26 red fluorescent cell linker midi kit, CellVue labeling kit, cholera toxin subunit B FITC conjugate, FITC conjugated 10 kDa dextran, FITC-conjugated transferrin, Phalloidin-TRITC, amiloride, methyl-β-cyclodextrin (MβCD), filipin III, nocodazole, puromycin dihydrochloride, G418, Cytochalasin D, and Lantrunculin A were all from Sigma Aldrich. Dynabeads® Protein G and Hoechst 33342 were from Invitrogen. SEA Block Blocking buffer was from Thermo Scientific. DiI-labeled acetylated LDL was from Harbor Bioproducts. Simvastatin was from Calbiochem. Complete protease inhibitors and phosphatase inhibitors were from Roche. Human phosphokinase antibody array (#ARY003) was from R&D Systems. U0126 was purchased from Selleck Chemicals. Plasmid encoding CD63-mCherry was kindly provided by Dr Lippincott-Schwartz and CD63-GFP plasmids was a gift from Dr. Takahisa Takino (Kanazawa University). The plasmid encoding pIRESneo-EGFP-α-tubulin was obtained from Addgene (Patricia Wadsworth, plasmid #12298). Plasmid encoding CAV1-YFP (pEX_EF1_CAV1-YFP) was from ATCC.

##### Exosome Purification and Characterization

Secreted vesicles were isolated from cell culture medium of U87 MG as previously described (20). Transmission electron microscopy was performed to validate the presence and purity of intact exosomes and analyzed for size using nanoparticle tracking analysis (NTA). Protein amounts were quantified with BCA^TM^ protein assay kit (Pierce) in each vesicle preparation. Isolated exosomes were in indicated experiments labeled with PKH67, PKH26 or cellvue midklaret fluorescent labeling kit according to the manufacturer's protocol (Sigma). Each vesicle preparation was stored at 4 °C and used within 5 days after isolation.

##### Confocal Laser Scanning Microscopy and Live-cell Imaging of Internalized Exosomes

All experiments were performed in Zeiss LSM 710 confocal scanning equipment using excitation wavelengths of 405, 488, 546, 633 nm, and a Plan-Apochromat 20×/0.8M27 objective and a C-Apochromat 63X/1.20W korr M27 glycerol immersion objective. Image analysis was performed using the Zeiss LSM software. Live cell imaging of internalized exosomes was performed with the same microscope equipped with heat incubator set at 37 °C with 5% CO_2_. For live cell confocal laser scanning microscopy experiments, cells were grown in glass bottom chamber slides, and 10–20 μg/ml of labeled exosomes were added to subconfluent cells in phenol red-free and serum-free conditions and incubated as indicated in the figure legend. Surface-bound exosomes were removed by extensive washing with 1 m NaCl and serum-free medium, followed by live cell imaging of intracellular exosomes in phenol-red free medium. For colocalization studies, 10 μg/ml CtxB-FITC, 100 μg/ml Dx10-FITC, or 150 μg/ml Tfn-FITC was co-incubated with labeled exosomes and incubated for 30 min. Cells were washed as above and fixed using 2% (w/v) paraformaldehyde for 5 min at room temperature. Cells were immediately analyzed. The weighted colocalization coefficients were calculated in representative cells using the Zeiss LSM software, and represent the number of red pixels (exosomes) that colocalize with turquoise pixels (CtxB or Dx10) divided by the total number of turquoise pixels.

##### TIRF Microscopy of Cell Surface-bound Exosomes

Total internal reflection fluorescence (TIRF) microscopy experiments were performed using an inverted microscope (Axio Observer Z1; Zeiss) equipped with a Zeiss TIRF module and a 100× 1.45 NA DIC M27 Zeiss TIRF oil immersion lens and acquisitions were made by using Slidebook 5.5 (3I). Subconfluent HeLa CAV1-YFP cells were incubated with labeled exosomes for 1 h in serum-free medium prior to live cell microscopy in phenol red-free medium.

##### Flow Cytometry Analysis

Subconfluent cells were incubated with labeled exosomes for the indicated time periods. Cells were washed in PBS, detached with trypsin, and subsequently washed twice in PBS supplemented with 1% BSA (w/v) and analyzed by flow cytometry on a FACS-Calibur instrument integrated with Cell-Quest software (BD Biosciences). Graphs show mean fluorescent values (10,000 events/sample) of one of three representative experiments (*n* = 3) with similar results ± S.D. unless stated otherwise.

##### Electron Microscopy

Isolated exosomes or cells incubated with or without exosomes were washed twice in Tris-buffered saline (TBS), pelleted, fixed for 1 h at 20 °C, and overnight at 4 °C in 2.5% glutaraldehyde in cacodylate buffer. For uptake of exosomes in HUVECs, cells were incubated with 10 μg/ml exosomes followed by trypsinization, washing in TBS and fixation in 2.5% glutaraldehyde in cacodylate buffer overnight at 4 °C. Some samples were incubated with the primary antibodies TF (10H10 titer 1:50) and tsg101 polyclonal (rabbit, titer 1:100) or flotillin-1 antibody (rabbit, 10 μg/ml) followed by detection with secondary antibodies conjugated with 5 nm gold, TF (1:10), or 15 nm gold (tsg101 and flotillin-1, titer 1:20) and analyzed using a JEOL JEM 1230 transmission electron microscope (JEOL, Peabody, MA) as previously described (20).

##### Transfections and Transductions

DNA plasmid transfections were performed in U87 MG cells (CD63-GFP), COS-7 (tubulin-GFP) or MEF, U87MG, CHO-K1, and HeLa (CAV1-YFP) seeded in chamber slides for confocal imaging or 24-well plates for flow cytometry analysis and grown in their respective medium w/o antibiotics. RNA interference was performed in HUVECs or U87 MG seeded in respective growth medium w/o antibiotics. All transfections were performed using Lipofectamine (Invitrogen) and 100 nm siRNA against CD63, HSP27, or clathrin following the recommendations of the manufacturer. U87 MG cells were transduced using Mission Lentiviral transduction particles (SHCLNV, Sigma Aldrich) for CAV1 (NM_001753 clone TRCN000008002) and negative control (negative control (SHCO16V, pLKO.1-puro)) at a MOI of 0.2 and selected by 1 μg/ml puromycin.

##### Immunoblotting and Immunoprecipitation

Cells or exosomes were washed with ice-cold PBS and lysed in a reducing RIPA buffer containing 20 mm Tris-HCl (pH 7.5), 150 mm NaCl, 1 mm Na_2_EDTA, 1 mm EGTA, 1% Nonidet P-40, 1% sodium deoxycholate, 2.5 mm sodium pyrophosphate, 1 mm β-glycerophosphate, 1 mm Na_3_VO_4_, 1 μg/ml leupeptin, and complete mini protease inhibitor mixture (Roche Diagnostics). For CD63 antibody requiring non-reduced conditions, samples were lysed in Triton-X buffer (20 mm Tris-HCl, pH 8.0, 137 mm NaCl, 1% (v/v) Triton X-100, 2 mm EDTA) supplemented with complete mini protease inhibitor mixture. Proteins were fractionated by electrophoresis, blotted, and developed using HRP substrate. The intensity of the bands was quantified using ImageJ software (NIH) using α-tubulin as loading control unless stated otherwise. For immunoprecipitation using magnetic Protein G® beads, cells were lysed in denaturing lysis buffer, and 500 μg of protein were immunoprecipitated using 1 μg of CAV1 antibody and incubated with rotation at 4 °C overnight. Resuspended Dynabeads were added (1.5 mg) and incubated with rotation at 4 °C for 3 h. Dynabeads®-Ab-antigen complexes were washed four times in PBS with Ca^2+^/Mg^2+^ before elution and further Western blotting analysis using 20% SEA Block in TBS supplemented with 1% (v/v) Tween20 as blocking buffer and for incubation with primary and secondary antibodies. Proteins were fractionated by electrophoresis, blotted, and developed using HRP substrate.

##### Phosphokinase Array

HUVECs were starved for 16 h in serum free medium and were either untreated or stimulated with exosomes (20 μg/ml) for 10 or 30 min for phosphokinase antibody array (#ARY003, R&D) before lysate collection. Levels of phosphorylated proteins (200 μg per sample) were analyzed in cell lysates according to the protocol provided by the manufacturer. Corresponding protein amounts were used as a control of phosphoproteins residing in exosomes. The arrays were quantified using ImageJ software (NIH). Values are expressed as the mean intensity relative to reference (Ref) of the respective blot.

##### Statistical Analysis

All data are presented as the mean of triplicates ± S.D. Statistical significance was evaluated with Student's two-tailed unpaired *t* test using Microsoft Excel. In some cases, the error bars were smaller than the symbols.

## RESULTS AND DISCUSSION

### 

#### 

##### Exosomes Enter Cells by Endocytosis and Travel Along Endosomal Cytoskeletal Routes

**FIGURE 1.**
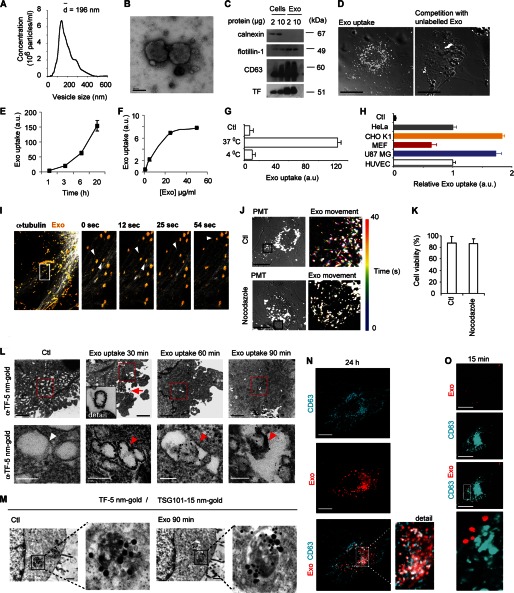
**Endocytosis of GBM cell-derived exosome-like extracellular vesicles.** *A*, characterization of U87 MG-derived vesicles by nanoparticle tracking analysis. *B*, electron microscopy validates intact vesicles. Scale bar, 100 nm. *C*, immunoblot analysis of cells and exosome-like vesicles for the exosomal markers CD63, TF, and flotillin-1, and the ER marker calnexin. *D*, confocal microscopy analysis of exosome uptake in the absence or presence of an excess (×4) unlabeled exosomes. Scale bars, 15 μm. *E* and *F*, time (*E*), and concentration (*F*)-dependent uptake of exosomes using flow cytometry analysis. *G*, insignificant passive uptake of exosomes at 4 °C. *H*, exosome uptake in human cervix adenocarcinoma cells (HeLa), chinese hamster ovary cells (CHO K1), mouse embryonic fibroblasts (MEF), U87 MG and HUVEC cells (*n* = 3). Values are normalized to HUVEC ( = 1). Control (*Ctl*) represents cells without exosomes. Flow cytometry graphs represent mean fluorescent values of one of two representative experiments generating similar results, error bars are ± S.D.; a.u., arbitrary units. *I*, exosomes move along microtubule tracks. COS-7 cells were transfected with pIRESneo-EGFP-α-tubulin 24 h prior to the addition of PKH26 labeled exosomes for an additional 16 h. Movie sequences display (4 boxed individual images, 0 s, 12 s, 25 s, 54 s) of exosome transport (*yellow*) along microtubule (*white*). *Arrowheads* depict intracellular, motile exosomes. Images were captured using a C-Apochromat 20X/0.8 M27 objective, 4.0 zoom, pinhole setting of 31 μm and laser gains of 5.5% (561 nm) and 5% (488 nm). Image size was x:512, y:512, and images were captured during 2 min and 11 s. Scale bar, 20 μm. For full-length movie, see supplemental video S2. *J*, reduced exosome transport in HUVECs treated for 10 min with 10 μg/ml nocodazole (*lower panels*) compared with Control (no treatment, *upper panels*). Pictures shown (Exo movement) represent color-coded data from time series of exosome movement in an overlay in which every time point (during 40 s) corresponds to a color. Images were captured using a C-Apochromat 63X/1.20W korr M27 objective (zoom 3.6). Scale bars, 50 μm. For full-length movies, see supplemental videos S3 and S4. *K*, cell viability is intact as measured by trypan blue exclusion after 10 min of nocodazole (10 μg/ml) treatment. *L*, electron microscopy images of compartments with internalized tissue factor (TF)-bearing exosomes over time. Low magnification overviews (*upper panels*, scale bars, 100 μm) confirm the intracellular localization of exosomes, and cropped pictures (*lower panels*, scale bars, 100 nm) demonstrate intraluminal vesicles (*red arrowheads*) positive for α-TF 5 nm gold particles. Note that endogenous vesicular structures in HUVECs are negative for TF (*lower left panel*, *white arrowhead*). *M*, electron microscopy colocalization studies in HUVECs of GBM cell-derived exosomes detected by α-TF 5 nm gold particles and MVBs distinguished by anti-TSG101 15 nm gold particles (scale bars, 100 μm). *Ctl*: no addition of exosomes. *N–O*, exosomes reside in a CD63-positive compartment after long-term incubation (24 h) but do not colocalize with CD63 at the cell surface. U87MG cells stably expressing CD63-mCherry (*N*) or transiently transfected with CD63-GFP (*O*) were incubated with PKH-labeled exosomes for the indicated time periods. Cells were washed in 1 m NaCl and PBS to remove nonspecifically bound exosomes before fixation and confocal microscopy analysis. CD63 (*turquoise*) and internalized exosomes (*red*) were captured at the indicated time points. Scale bars, 15 μm.

The GBM cell line U87 MG secretes exosome-like microvesicles or exosomes previously characterized by us and others for features and function (9–11, 20). Nanotracking analysis and electron microscopy showed 50–400 nm-sized vesicles (Fig. 1, *A* and *B*) that were further characterized for the presence of the exosomal markers CD63, Tissue Factor (TF), and flotillin-1 and the absence of the ER marker calnexin, demonstrating purity of vesicles (Fig. 1*C*). The internalization of exosomes was visualized by confocal fluorescence microscopy in human umbilical vein endothelial cells (HUVECs) (Fig. 1*D*, *left panel*) and U87 MG cells (supplemental video S1). The specificity of the uptake of fluorescent exosomes was supported by efficient attenuation by an excess of unlabeled exosomes (Fig. 1*D*, *right panel*).

The uptake mechanism of exosomes has been a matter of debate, *i.e.* whether exosomes enter cells through direct fusion with the plasma membrane of recipient cells or through endocytosis (18, 19). Here, exosomes displayed time and concentration-dependent uptake kinetics (Fig. 1, *E* and *F*), and incubation at 4 °C efficiently attenuated uptake, suggesting an energy-dependent process rather than passive membrane passage (Fig. 1*G*). Further, several normal and transformed cell-lines were able to internalize exosomes at significant levels (Fig. 1*H*), which argues against that exosome transfer is restricted to specific cell-types as has previously been suggested (17). Live confocal imaging with tubulin-GFP-expressing cells revealed that internalized exosomes travel along microtubules following their uptake (Fig. 1*I* and supplemental video S2). The mobility of internalized exosomes was substantially reduced by interference with microtubule polymerization using nocodazole (Fig. 1*J* and supplemental videos S3 and S4). Importantly, under these conditions nocodazole had no unspecific effect on cell viability (Fig. 1*K***).** These data are consistent with an endocytic process rather than membrane fusion as exosomes followed a time, concentration, and temperature-dependent pathway. The fact that disruption of microtubule attenuated intracellular exosome transport further indicates that exosomes are not fused with the plasma membrane, but rather are dependent on the regular endosomal transportation machinery for further intracellular sorting. To corroborate these data, tissue factor (TF), previously shown to reside in GBM cell-derived exosomes while absent in HUVECs (20), was used to discriminate between endogenous vesicles and internalized exosomes in electron microscopy analysis. Immunolabeling for TF demonstrated that internalized exosomes are enclosed in double membrane structures and do not merge with the plasma membrane of recipient HUVECs (Fig. 1*L*).

Exosomes were shown to be sorted to larger compartments positive for the MVB marker TSG101 (tumor susceptibility gene 101 protein) (22) (Fig. 1*M*). In further support of the sorting of endocytosed exosomes to MVBs, a substantial fraction of internalized vesicles were located in a CD63 (LAMP-3, Tspn30)-positive compartment (Fig. 1*N*). Experiments with exosomes derived from CD63-GFP transfected cells pre-incubated with PKH-labeled exosomes, suggested that mixing of exosomal constituents can occur during maturation in MVBs, resulting in the generation of compound vesicles (supplemental Fig. S1, *A* and *B*). However, siRNA-mediated knockdown of CD63 or antibody-mediated cell-surface blocking of CD63 did not significantly affect exosome uptake (supplemental Fig. S1, *C–E*). These results, and the fact that exosomes did not colocalize with CD63 at short incubation times (Fig. 1*O*), suggest that although exosomes are sorted to CD63-positive vesicles in recipient cells, CD63 has no direct role in the endocytic uptake of exosomes.

##### Role of Lipid Raft-mediated Endocytosis in Exosome Uptake

**FIGURE 2.**
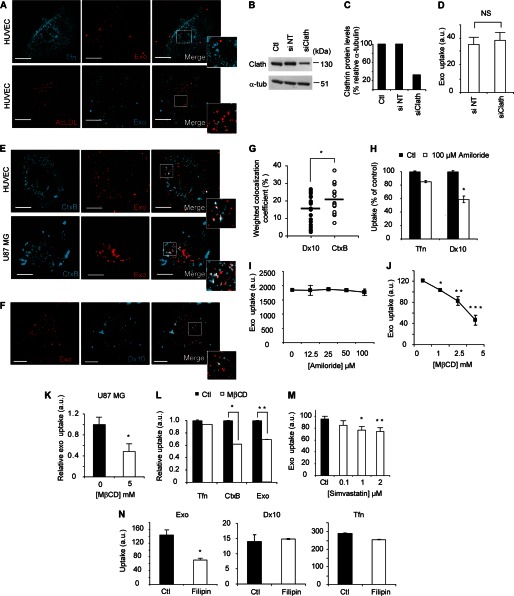
**Endocytic uptake of exosomes requires intact lipid membrane rafts.** *A*, confocal microscopy analysis shows no colocalization of Tfn (*turquoise*, *upper panel*) or AcLDL (*red*, *lower panel*) with exosomes (*red*/*turquoise*) at 30 min. Scale bars, 15 μm. *B*, knockdown validation of clathrin heavy chain using siRNA against clathrin (*si Clath*) as compared with negative control sequence (*si NT*) and normalized to α-tubulin (*C*). *D*, flow cytometry analysis of exosome uptake shows no difference in si Clath as compared with si NT-transfected cells. *E*, confocal microscopy analysis shows colocalization of CtxB (turquoise) and exosomes (*red*) at 30 min of uptake in HUVECs (*upper panel*) and U87 MG cells (*lower panel*). Scale bars, 15 μm. *F*, confocal microscopy analysis shows limited colocalization of Dx10 (*turquoise*) and exosomes (*red*) at 30 min of uptake in HUVECs. Scale bars, 15 μm. *G*, weighted colocalization coefficients display 20% (mean value) colocalization of CtxB and exosomes, and ∼15% for exosomes and 10 kDa dextran (Dx10). Colocalization coefficients were calculated (Dx10/exosomes, *n* = 23 cells; CtxB/exosomes, *n* = 22 cells) using Zeiss Zen software. *, *p* = 0.0182. All images were captured using a C-Apochromat 63X/1.20W korr M27 objective using laser gain of 6.0% in both lasers. *H*, macropinocytosis inhibitor amiloride (100 μm) decreases Dx10uptake (*, *p* = 0.01) while Tfn uptake is less affected. *I*, amiloride has no significant effect on exosome uptake at a wide range of concentrations. *J* and *K*, cholesterol-depleting drug MβCD dose-dependently inhibits exosome uptake. *J*, HUVECs (*p* values, *, 0.005, **, 0.0023, ***, 0.0008); *K*, U87 MG cells (*, *p* = 0.068). *L*, uptake of Tfn is not affected by MβCD (2.5 mm), while CtxB and exosome uptake are reduced, suggesting specific inhibition of lipid raft-dependent uptake (*p* values, *, 0.0006, **, 0.02). *M*, simvastatin dose-dependently inhibits exosome uptake (*p* values, *, 0.014, **, 0.011). *N*, sequestration of lipid rafts by filipin III substantially inhibits exosome uptake (*left panel*) while Dx10 (*middle panel*) and Tfn (*right panel*) uptake are less affected (*, *p* = 0.001). Presented graphs show mean fluorescent values (10,000 events/sample) of one of three representative experiments (*n* = 3) with similar results ± S.D.

We next sought to define the distinct, cellular pathways associated with the endocytic uptake of exosomes. We found no colocalization of exosomes either with transferrin (Tfn) or acetylated LDL (Fig. 2*A*), *i.e.* conventional ligands of classical, clathrin-dependent endocytosis (2). Accordingly, 80% knockdown of clathrin (heavy chain) (Fig. 2, *B* and *C*) had no effect on exosome uptake (Fig. 2*D*). Interestingly, when evaluating by confocal microscopy the colocalization coefficient of exosomes and the lipid raft marker cholera toxin subunit B (CtxB) at short-term incubation, we found ∼20% colocalization (Fig. 2, *E* and *G*). CtxB binds strongly to GM1 (monosialotetrahexosylganglioside), an established constituent of membrane lipid rafts, the integrity of which depends on the high abundance of cholesterol. We found some colocalization of exosomes and the macropinocytosis/fluid-phase marker 10 kDa dextran (Dx10); however, significantly less than between exosomes and CtxB (Fig. 2, *F* and *G*). Amiloride, *i.e.* an inhibitor of Na^+^/H^+^ exchange important for macropinocytotic uptake expectedly inhibited Dx10 uptake (Fig. 2*H*); however, amiloride had no significant effect on exosome uptake at any concentration tested (Fig. 2*I*). In support of a role of membrane rafts, exosome uptake was highly sensitive to membrane cholesterol depletion by MβCD. Exosome internalization was inhibited by MβCD in a dose-dependent manner, and at the highest concentration used ∼60% reduction of uptake was shown in HUVECs (Fig. 2*J*). Importantly, this effect was not restricted to HUVECs, as MβCD similarly inhibited exosome uptake in U87 MG cells (Fig. 2*K*). The uptake of fluorescently labeled Tfn was intact while CtxB uptake was expectedly inhibited (Fig. 2*L*), confirming that MβCD preferentially inhibits non-clathrin dependent endocytosis under these conditions. MβCD could potentially also disrupt cholesterol-rich domains of the exosomal membrane. We therefore performed experiments with a statin (simvastatin), which acts through inhibition of the rate-limiting enzyme of cholesterol biosynthesis, 3-hydroxyl-3-methylglutaryl coenzyme A (HMG-CoA) reductase, and that is widely used as a cholesterol lowering drug in humans. Notably, simvastatin was previously shown to reduce intracellular cholesterol levels in HUVECs (23). We show that simvastatin can dose-dependently inhibit exosome internalization in these cells (Fig. 2*M*).

Non-classical endocytosis pathways (also defined as lipid raft associated pathways) can be either dependent or independent of CAV1 (2, 4). Caveolae-dependent endocytosis is a well studied clathrin-independent pathway and shares many features with membrane lipid rafts (24, 25). Importantly, filipin III, an inhibitor of lipid raft-dependent and caveolar endocytosis, was shown to inhibit exosome uptake by ∼50% (Fig. 2*N*, *left panel*). As controls of filipin specificity, the uptake of Dx10 and Tfn was almost unaffected (Fig. 2*N*, *middle* and *right panels*).

##### CAV1 Negatively Regulates Exosome Uptake

**FIGURE 3.**
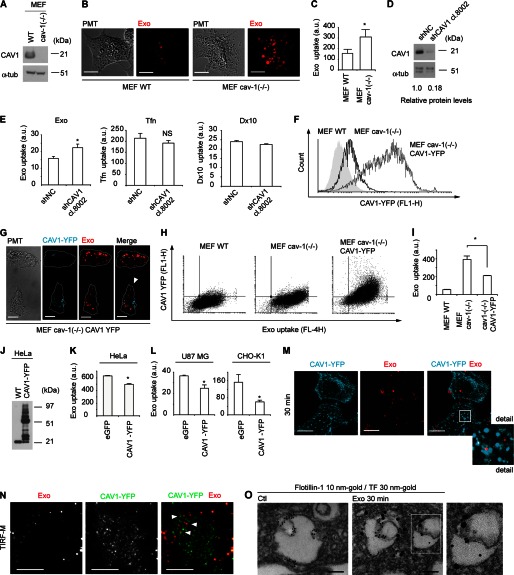
**Exosome internalization is negatively regulated by CAV1.** *A*, mouse embryonic fibroblasts from wild-type (*MEF WT*) and cav-1 knock-out mice (MEF cav-1(−/−)) were analyzed for CAV1 protein. *B*, confocal images show elevated uptake of exosomes (*red*) in MEF cav-1 (−/−) as compared with MEF WT cells. Scale bars, 15 μm. *C*, graph shows quantitative measurement of exosome uptake in MEF WT and MEF cav-1 (−/−) cells by flow cytometry, and represents mean fluorescent values (20,000 events/sample) of three independent experiments (*n* = 9) ± S.D. (*, *p* = 0.000003). *D*, stable knockdown (by ∼80%) of CAV1 by lentiviral shRNA transduction in U87 MG cells. *E*, flow cytometry analysis demonstrates increased exosome uptake (*left panel*), no significant difference in Tfn uptake (*middle panel*), and decreased uptake of Dx10 (*right panel*) in CAV1 shRNA cells, as compared with cells transfected with control shRNA (shRNA NC). Graph represents mean fluorescent values (20,000 events/sample) of one of three representative experiments (*n* = 4) ± S.D. (*, *p* = 0.0019 in *left panel*). *F*, flow cytometry analysis of CAV1-YFP transfection efficiency; MEF WT (*gray area*), MEF cav-1 (−/−) (*black line*), and MEF cav-1 (−/−) cells transfected with pEX_EF1_CAV1-YFP plasmid (*gray line*). *G*, introduction of CAV1-YFP in MEF cav-1 (−/−) cells (YFP, *turquoise*) reduces uptake of exosomes (*red*). Note that the high CAV1-YFP-expressing cell (*arrowhead*) displays reduced exosome uptake as compared with the low CAV1-YFP expressing cell. Scale bars, 15 μm. *H*, dot plot analysis of exosome uptake *versus* CAV1-YFP expression in MEF cells, as indicated. *I*, quantification of the uptake in *H*. Graph represents mean fluorescent values (20,000 events/sample) of a representative experiment (*n* = 4) ± S.D. (*, *p* = 0.0009). *J*, immunoblotting for CAV1 in HeLa cells stably transfected with CAV1-YFP. *K*, reduced uptake of exosomes in CAV1 overexpressing HeLa cells as compared with HeLa WT cells. *L*, reduced uptake of exosomes in transiently CAV1 overexpressing U87 MG cells (*left panel*) and CHO-K1 cells (*right panel*) as compared with control cells transiently transfected with eGFP. Graph represents mean fluorescent values (20,000 events/sample) of one out of two independent experiments (*n* = 3) ± S.D. (*, *p* = 0.018). *M*, no colocalization between CAV1-YFP (turquoise) and exosomes (*red*) using confocal microscopy analysis. Scale bars, 15 μm. *N*, no colocalization between CAV1-YFP (*green*) and exosomes (*red*) using TIRF microscopy analysis. Scale bars, 10 μm. *O*, electron microscopy colocalization studies in HUVECs of exosomes detected by α-TF 30 nm gold particles and lipid rafts distinguished by anti-flotillin-1 10 nm gold particles. Scale bars, 100 μm. *Left panel*: *Ctl*, no addition of exosomes.

The above data prompted further studies on the role of CAV1 in endocytic uptake of exosomes. Intriguingly, CAV1 knock out cells (MEF cav 1 (−/−)) (Fig. 3*A*) displayed increased levels of exosome uptake as compared with wild type cells (MEF cav 1 (+/+), here denoted as MEF WT) as demonstrated by confocal microscopy (Fig. 3*B***)** and flow cytometry analyses (Fig. 3*C*). Somewhat unexpectedly, these results suggested a negative regulatory role of CAV1 in exosome uptake. This was also true for U87 MG cells, as stable knockdown of CAV1 (Fig. 3*D*) resulted in significantly increased uptake of exosomes (Fig. 3*E*, *left panel*). As important controls, knockdown of CAV1 did not significantly alter the uptake of clathrin-mediated endocytosis, and macropinocytosis was slightly decreased, as evaluated by Tfn and Dx10 uptake, respectively (Fig. 3*E*, *middle* and *right panels*). Moreover, rescue experiments in which CAV1-YFP was ectopically expressed in MEF cav 1 (−/−) (Fig. 3*F*), showed reduced exosome uptake by ∼50% as compared with control MEF cav 1 (−/−) cells (Fig. 3, *G–I*). In line with these results, HeLa cells stably transfected to highly overexpress CAV1-YFP exhibited significantly reduced uptake of exosomes (Fig. 3, *J* and *K*). We could show that these effects were not restricted to MEF and HeLa cells, as U87 MG and CHO-K1 cells transfected with CAV1-YFP plasmid displayed significantly reduced exosome uptake as compared with control plasmid transfected cells (Fig. 3*L*).

It has previously been shown that endocytic uptake of virus particles is negatively regulated by CAV1 by stabilization of specific plasma membrane lipid raft domains (1, 26, 27). We next employed confocal microscopy co-localization studies of CAV1-YFP and PKH-labeled exosomes. As shown in Fig. 3*M*, we could not detect any colocalization at 30 min of incubation, suggesting that exosomes are not associated with CAV1-positive endosomal structures. Confocal microscopy cannot fully discriminate between newly internalized and cell-surface associated exosomes, *i.e.* the above data do not exclude the possibility of exosomal association with CAV1-containing lipid rafts at earlier time points than 30 min. We therefore applied TIRF microscopy to visualize exosomes present in the membrane region close to the cell surface, and during early phases of internalization. These analyses confirmed that exosomes do not appear to colocalize with CAV1 upon internalization (Fig. 3*N*). Together, these findings indicate that the negative regulatory function of CAV1 does not occur through stabilization of lipid raft domains directly involved in exosome uptake. To corroborate confocal microscopy data, showing that internalized exosomes are associated with lipid rafts (Fig. 2*E***)**, we next performed immunoelectron microscopy colocalization studies of exosomes (by tissue factor antibody staining to discriminate from endogenous endosomes) and the lipid raft marker flotillin-1. We found a substantial colocalization at 30 min of internalization, signifying that exosomes indeed are taken up through lipid rafts (Fig. 3*O*).

##### Role of ERK1/2-HSP27 Signaling Activation in Exosome Uptake

**FIGURE 4.**
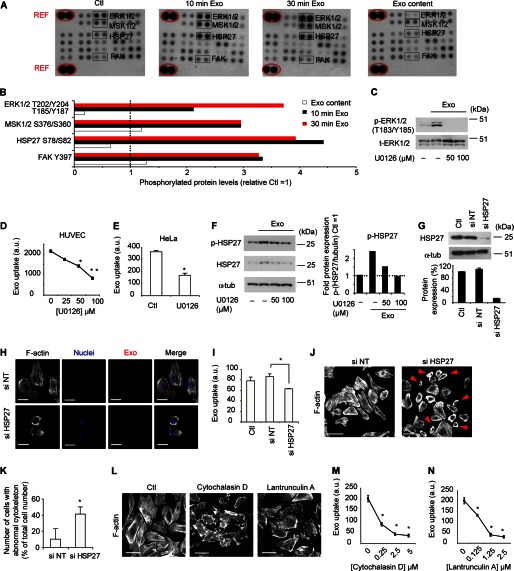
**Exosome internalization depends on ERK1/2 and HSP27 signaling activation and an intact cytoskeleton.** *A*, levels of phosphorylated kinases in HUVECs with no treatment (*Ctl*), or incubated with exosomes for 10 min (*10 min Exo*) or 30 min (*30 min Exo*). As a comparison, same protein amount of exosomes was used to visualize phosphoproteins residing in exosomes (*Exo content*). *B*, quantification of the mean value (*n* = 2 in each blot) of p-ERK1/2, p-MSK1/2, p-HSP27, and p-FAK relative unstimulated cells (*Ctl*). *C*, relative protein levels of p-ERK1/2 with or without exosome stimulation in the absence or presence of U0126. *D*, reduced exosome uptake in HUVECs treated with U0126. Graphs represents mean fluorescent values (20,000 events/sample) of one of three independent experiments (*n* = 3) (*, *p* = 0.01, **, *p* = 0.002) ± S.D. *E*, reduced exosome uptake in HeLa cells treated with U0126, expressed as mean fluorescent values (20,000 events/sample) of one of two independent experiments (*n* = 4) (*, *p* = 0.000009) ± S.D. *F*, induction of p-HSP27 protein by exosomes is counteracted by ERK1/2 inhibition using U0126. *G*, representative blot of relative HSP27 protein expression after siRNA knockdown. *H*, reduced exosome (*red*) uptake in HSP27 knockdown cells. *White*, f-actin; *blue*, nuclei. Scale bars, 15 μm. *I*, flow cytometry analysis of cells in *H* from a representative experiment (*n* = 4) (*, *p* = 0.002) ± S.D. *J*, actin cytoskeleton phalloidin stainings in HSP27 siRNA and siRNA NT-transfected cells. Note the abnormal cytoskeleton in siHSP27-transfected cells (*right panel*, *red arrowheads*). Scale bars, 100 μm. *K*, quantification of the average number of cells with abnormal cytoskeleton relative the total number of cells counted in seven independent microscopic fields; siNT cells (*n* = 116) and siHSP27 knockdown cells (*n* = 209). Data are mean values ± S.D. (*, *p* = 0.00018). *L*, disruption of the actin cytoskeleton (f-actin, *white*) using 0.5 μm Cytochalasin D or Lantrunculin A. Scale bars, 100 μm. *M* and *N*, exosome uptake in cells treated with varying concentrations of Cytochalasin D (*M*) or Lantrunculin A (*N*). Graphs are mean fluorescent values (10,000 events/sample) of two independent experiments with similar results ± S.D. All values (*) were significantly different from control (0 μm) with a *p* value of < 0.0001.

Our findings that CAV1 negatively regulates membrane raft-dependent exosome uptake without apparent co-localization with exosomes, prompted further mechanistic studies on the role of CAV1. Apart from its role as a structural component of lipid rafts, CAV1 has been shown to modify the activity of several signaling proteins, such as Src family members, epidermal growth factor receptor, and integrins (28–30). Consequently, we set out to explore potential signaling mechanisms involved in exosome uptake. In initial experiments, using phospho-kinase arrays, we found that short-term incubation with exosomes resulted in 2–4.5-fold induction of several lipid raft associated proteins; p-FAK, the heat-shock protein p-HSP27, and p-ERK1/2 and its downstream target p-MSK1/2 (Fig. 4, *A* and *B*) (for a complete list of relative phospho-kinase levels, see supplemental Fig. S2). Exosomal induction of p-ERK1/2 and p-HSP27 were validated by Western blotting (Fig. 4, *C* and *F*). The mitogen-activated protein kinase (MAPK) pathway may be initiated at the cell surface and continue during endosomal sorting, while more recent studies suggest that MAPK signaling is a required element of endocytosis (31). Interestingly, pharmacological targeting of ERK1/2 signaling using the specific inhibitor.

U0126 (Fig. 4*C*) dose-dependently decreased exosome uptake in HUVECs (Fig. 4*D*) as well as in HeLa cells (Fig. 4*E*). We next turned our interest to exosome-dependent induction of p-HSP27 (Fig. 4, *A* and *B*) as HSPs besides from being localized in the cytosol, have been associated with the cellular membrane and lipid rafts (32). Accordingly, previous reports have shown a role of p-HSP27 in macromolecular internalization by regulation of actin cytoskeleton dynamics (33, 34). We found that inhibition of ERK1/2 signaling dose-dependently reduced p-HSP27 levels (Fig. 4*F*), suggesting that, at least in the context of exosome uptake, HSP27 is activated downstream of ERK1/2. A direct role of HSP27 as an effector molecule in exosome internalization was supported by significantly reduced exosome uptake upon siRNA-mediated knock-down of HSP27 (Fig. 4, *H* and *I*). In agreement with the previously understood role of HSP27 in cytoskeleton rearrangement, cells transfected with siRNA for HSP27 as compared with non-target siRNA exhibited an abnormal actin cytoskeleton as assessed by phalloidin staining (Fig. 4, *J* and *K*). Pharmacological disruption of the actin cytoskeleton (Fig. 4*L*) using Cytochalasin D or Lantrunculin A inhibited uptake of exosomes under similar conditions (Fig. 4, *M* and *N*), reinforcing the notion that an intact actin cytoskeleton is required for efficient exosome uptake. We conclude that exosomes may trigger lipid raft mediated endocytosis through signaling activation of ERK1/2-dependent pathways that include a specific role of HSP27.

##### CAV1 Regulates Uptake of Exosomes through Suppression of ERK1/2 Signaling

**FIGURE 5.**
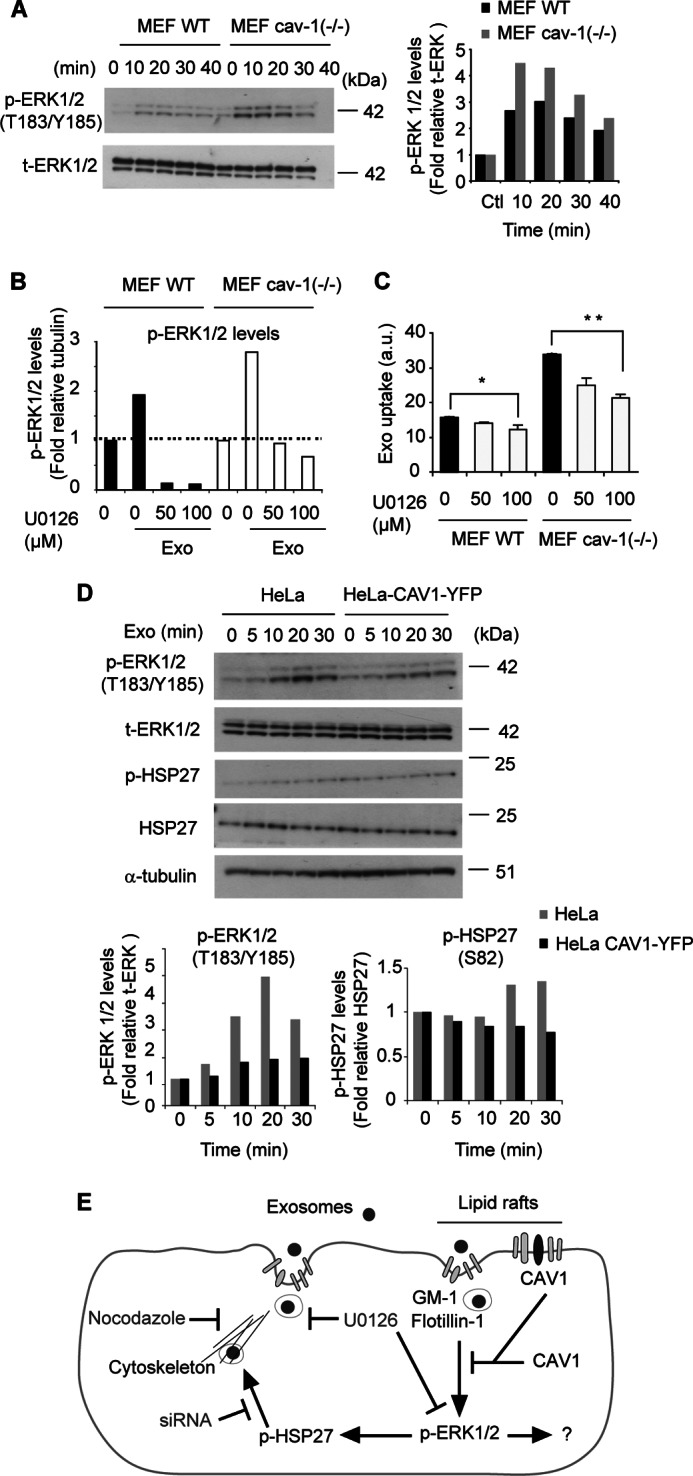
**CAV1 negatively regulates ERK1/2-dependent endocytosis of exosomes.** *A*, shown is a representative blot for p-ERK1/2 and total ERK1/2 (t-ERK1/2) in MEF WT and MEF cav-1 (−/−) cells stimulated with exosomes for the indicated times (*left panel*), and quantification of relative protein levels (*right panel*). *B*, reversal of exosome-mediated induction of p-ERK1/2 by U0126 in MEF cells. Graph shows quantification of Western blot analysis. *C*, up-regulation of exosome uptake in CAV1-deficient cells is counteracted by ERK1/2 inhibition. Graph represents mean fluorescent values (20,000 events/sample) of one of three independent experiments with similar results ± S.D. *, *p* = 0.02, **, *p* = 0.00001. *D*, overexpression of CAV1-YFP suppresses exosome-mediated induction of p-ERK1/2 and p-HSP27 in HeLa cells. Shown are representative blots for p-ERK1/2, t-ERK1/2, p-HSP27, total HSP27, and tubulin (*upper panel*), and quantification of relative protein levels (*lower panel*) in HeLa WT and CAV1-YFP cells stimulated with exosomes for the indicated times. *E*, schematic figure of the major findings of the present work. Exosomes are internalized by lipid raft-associated endocytosis, which is under negative control by CAV1. Additional signaling proteins involved are ERK1/2 and HSP27, and probably additional ERK1/2 downstream targets. HSP27 is known to be involved in rearrangement of the actin cytoskeleton important for the invagination of the plasma membrane during endocytosis.

From the above findings, we next explored the hypothesis that CAV1 negatively regulates exosome uptake through interference with ERK1/2 signaling. We could appreciate that the induction of p-ERK1/2 during exosome uptake was enhanced in MEF cav-1 (−/−) as compared with MEF WT cells at all time points tested (Fig. 5*A*). Previous studies suggested CAV1-mediated down-regulation of MAPK signaling in rodents but not in human fibroblasts (35); however, in the context of exosome uptake we observed CAV1-mediated suppression of p-ERK1/2 induction in both mouse (MEF) and human (HeLa)-derived cells (Fig. 5, *A* and *D*). Consistent with the findings in HUVECs (Fig. 4*D*) and HeLa cells (Fig. 4*E*), reduced levels of p-ERK1/2 by U0126 treatment in MEF cells (Fig. 5*B*) resulted in decreased internalization of exosomes (Fig. 5*C*). More importantly, enhanced exosome uptake by CAV1-defiency could be directly linked to ERK1/2 signaling, as U0126 treatment efficiently counteracted exosome uptake in MEF cav-1 (−/−) cells (Fig. 5*C*). Accordingly, in HeLa-CAV1-YFP overexpressing as compared with wild-type HeLa cells, we found reduced capability of exosomes to induce p-ERK1/2 signaling and subsequent p-HSP27 induction at multiple time points of exosome stimulation (Fig. 5*D*). We conclude that exosome uptake commences through lipid raft-mediated endocytosis associated with and dependent on signaling activation of ERK1/2 and HSP27. Further, our data indicate that CAV1 localized in the plasma membrane negatively regulates endocytic uptake of exosomes at least partly through suppression of ERK1/2 signaling activation (for a schematic overview, see Fig. 5*E*).

Here, we present several significant findings that advance our understanding of exosome uptake at the level of membrane uptake pathways and signaling regulation. First, we establish that exosome uptake mainly occurs through non-clathrin dependent, lipid raft-mediated endocytosis. To circumvent limitations caused by the high sensitivity of labeled exosomes to detergents and fixation solutions, we have in our studies applied live microscopy using fluorescent fusion protein constructs, fluorophore-labeled ligands and/or mild fixation procedures in combination with electron microscopy studies. Data from these studies strongly suggest that exosomes are internalized by endocytosis rather than by membrane fusion at the plasma membrane. More importantly, we identify specific signaling mechanisms implicated in the uptake process, and find an unexpected and significant role of CAV1 in regulating exosome uptake. Our data suggest that negative regulation of endocytic uptake of exosomes occurs through stabilization of lipid rafts by CAV1. However, these results do not exclude multilevel signaling through the described or other pathways regulated by or independent of CAV1. Further studies investigating other related pathways are of high interest.

Although we provide convincing evidence that ERK1/2 is activated by exosomes especially in the context of CAV1 deficiency, and that ERK1/2 activity is required for efficient exosome uptake, our studies do not fully elucidate the protein interactions involved. A reciprocal regulation of ERK1/2 and CAV1 has previously been described in other systems, where the activation of ERK1/2 was suppressed by CAV1, and the up-regulation of ERK1/2 in turn was shown to suppress CAV1 mRNA levels (36). Moreover, ERK1/2 was shown to localize to caveolae in the plasma membrane, and the caveolar localization of ERK1/2 negatively regulated further signal transduction to the nucleus (36, 37). In the context of exosome internalization, however, we failed to demonstrate a direct protein interaction of CAV1 and ERK1/2, as determined by confocal microscopy analyses and anti-CAV1 antibody pulldown experiments (supplemental Fig. S3, *A* and *B*). The protein interactions responsible for CAV1-mediated negative regulation of ERK1/2 signaling during exosome uptake remains has to be determined in future studies. Based on our findings of a role for CAV1 and ERK1/2 in exosome internalization, it may be speculated that uptake of extracellular vesicles is governed by the signaling status of recipient cells. In the context of the tumor microenvironment, this may be determined by specific oncogenetic events in malignant cells and the availability of *e.g.* growth factors, cytokines, and their respective receptors in the stromal compartment. Thus, future studies should explore the possibility of differential transfer of vesicles in the context of CAV1 and ERK1/2 expression, as these proteins are frequently deregulated at various stages of tumor development (38, 39). Moreover, as exosome-based delivery of mRNA and miRNA has been proposed as a feasible, therapeutic approach in cancer and other pathological conditions (40), further comprehensive understanding of vesicular uptake mechanisms should offer a more rational design of exosomes as drug delivery vehicles.

## Footnotes

2 The abbreviations used are:

MVB: multivesicular body
GBM: glioblastoma
CAV: caveolin
HSP: heat shock protein
NTA: nanoparticle tracking analysis
TIRF: total internal reflection fluorescence
TF: tissue factor
Tfn: transferrin
Ctx: cholera toxin
MβCD: methyl-β-cyclodextrin
HMG-CoA: 3-hydroxyl-3-methylglutaryl coenzyme A.

## References

[1] Damm E. M. Pelkmans L. Kartenbeck J. Mezzacasa A. Kurzchalia T. Helenius A. (2005) Clathrin- and caveolin-1-independent endocytosis: entry of simian virus 40 into cells devoid of caveolae. J. Cell Biol. 168, 477–488 15668298 10.1083/jcb.200407113 PMC2171728

[2] Doherty G. J. McMahon H. T. (2009) Mechanisms of endocytosis. Annu. Rev. Biochem. 78, 857–902 19317650 10.1146/annurev.biochem.78.081307.110540

[3] Parton R. G. Richards A. A. (2003) Lipid rafts and caveolae as portals for endocytosis: new insights and common mechanisms. Traffic 4, 724–738 14617356 10.1034/j.1600-0854.2003.00128.x

[4] Mayor S. Pagano R. E. (2007) Pathways of clathrin-independent endocytosis. Nat. Rev. Mol. Cell Biol. 8, 603–612 17609668 10.1038/nrm2216 PMC7617177

[5] Belting M. Wittrup A. (2008) Nanotubes, exosomes, and nucleic acid-binding peptides provide novel mechanisms of intercellular communication in eukaryotic cells: implications in health and disease. J. Cell Biol. 183, 1187–1191 19103810 10.1083/jcb.200810038 PMC2606965

[6] MacKenzie A. Wilson H. L. Kiss-Toth E. Dower S. K. North R. A. Surprenant A. (2001) Rapid secretion of interleukin-1β by microvesicle shedding. Immunity 15, 825–835 11728343 10.1016/s1074-7613(01)00229-1

[7] Baroni M. Pizzirani C. Pinotti M. Ferrari D. Adinolfi E. Calzavarini S. Caruso P. Bernardi F. Di Virgilio F. (2007) Stimulation of P2 (P2X7) receptors in human dendritic cells induces the release of tissue factor-bearing microparticles. FASEB J. 21, 1926–1933 17314141 10.1096/fj.06-7238com

[8] Mathivanan S. Ji H. Simpson R. J. (2010) Exosomes: extracellular organelles important in intercellular communication. J. Proteomics. 73, 1907–1920 20601276 10.1016/j.jprot.2010.06.006

[9] Skog J. Würdinger T. van Rijn S. Meijer D. H. Gainche L. Sena-Esteves M. Curry W. T. Jr. Carter B. S. Krichevsky A. M. Breakefield X. O. (2008) Glioblastoma microvesicles transport RNA and proteins that promote tumour growth and provide diagnostic biomarkers. Nature Cell Biol. 10, 1470–1476 19011622 10.1038/ncb1800 PMC3423894

[10] Al-Nedawi K. Meehan B. Micallef J. Lhotak V. May L. Guha A. Rak J. (2008) Intercellular transfer of the oncogenic receptor EGFRvIII by microvesicles derived from tumour cells. Nature Cell Biol. 10, 619–624 18425114 10.1038/ncb1725

[11] Al-Nedawi K. Meehan B. Kerbel R. S. Allison A. C. Rak J. (2009) Endothelial expression of autocrine VEGF upon the uptake of tumour-derived microvesicles containing oncogenic EGFR. Proc. Natl. Acad. Sci. U.S.A. 106, 3794–3799 19234131 10.1073/pnas.0804543106 PMC2656159

[12] Peinado H. Alečković M. Lavotshkin S. Matei I. Costa-Silva B. Moreno-Bueno G. Hergueta-Redondo M. Williams C. García-Santos G. Ghajar C. Nitadori-Hoshino A. Hoffman C. Badal K. Garcia B. A. Callahan M. K. Yuan J. Martins V. R. Skog J. Kaplan R. N. Brady M. S. Wolchok J. D. Chapman P. B. Kang Y. Bromberg J. Lyden D. (2012) Melanoma exosomes educate bone marrow progenitor cells toward a pro-metastatic phenotype through MET. Nature Med. 18, 883–891 22635005 10.1038/nm.2753 PMC3645291

[13] Gruenberg J. Stenmark H. (2004) The biogenesis of multivesicular endosomes. Nat. Rev. Mol. Cell Biol. 5, 317–323 15071556 10.1038/nrm1360

[14] Antonyak M. A. Li B. Boroughs L. K. Johnson J. L. Druso J. E. Bryant K. L. Holowka D. A. Cerione R. A. (2011) Cancer cell-derived microvesicles induce transformation by transferring tissue transglutaminase and fibronectin to recipient cells. Proc. Natl. Acad. Sci. U.S.A. 108, 4852–4857 21368175 10.1073/pnas.1017667108 PMC3064359

[15] Ostrowski M. Carmo N. B. Krumeich S. Fanget I. Raposo G. Savina A. Moita C. F. Schauer K. Hume A. N. Freitas R. P. Goud B. Benaroch P. Hacohen N. Fukuda M. Desnos C. Seabra M. C. Darchen F. Amigorena S. Moita L. F. Thery C. (2010) Rab27a and Rab27b control different steps of the exosome secretion pathway. Nature Cell Biology 12, 19–30 10.1038/ncb2000 19966785

[16] Baietti M. F. Zhang Z. Mortier E. Melchior A. Degeest G. Geeraerts A. Ivarsson Y. Depoortere F. Coomans C. Vermeiren E. Zimmermann P. David G. (2012) Syndecan-syntenin-ALIX regulates the biogenesis of exosomes. Nature Cell Biol. 14, 677–685 22660413 10.1038/ncb2502

[17] Feng D. Zhao W. L. Ye Y. Y. Bai X. C. Liu R. Q. Chang L. F. Zhou Q. Sui S. F. (2010) Cellular internalization of exosomes occurs through phagocytosis. Traffic 11, 675–687 20136776 10.1111/j.1600-0854.2010.01041.x

[18] Del Conde I. Shrimpton C. N. Thiagarajan P. López J. A. (2005) Tissue-factor-bearing microvesicles arise from lipid rafts and fuse with activated platelets to initiate coagulation. Blood 106, 1604–1611 15741221 10.1182/blood-2004-03-1095

[19] Parolini I. Federici C. Raggi C. Lugini L. Palleschi S. De Milito A. Coscia C. Iessi E. Logozzi M. Molinari A. Colone M. Tatti M. Sargiacomo M. Fais S. (2009) Microenvironmental pH is a key factor for exosome traffic in tumour cells. J. Biol. Chem. 284, 34211–34222 19801663 10.1074/jbc.M109.041152 PMC2797191

[20] Svensson K. J. Kucharzewska P. Christianson H. C. Sköld S. Löfstedt T. Johansson M. C. Mörgelin M. Bengzon J. Ruf W. Belting M. (2011) Hypoxia triggers a proangiogenic pathway involving cancer cell microvesicles and PAR-2-mediated heparin-binding EGF signaling in endothelial cells. Proc. Natl. Acad. Sci. U.S.A. 108, 13147–13152 21788507 10.1073/pnas.1104261108 PMC3156184

[21] Wittrup A. Zhang S. H. Svensson K. J. Kucharzewska P. Johansson M. C. Mörgelin M. Belting M. (2010) Magnetic nanoparticle-based isolation of endocytic vesicles reveals a role of the heat shock protein GRP75 in macromolecular delivery. Proc. Natl. Acad. Sci. U.S.A. 107, 13342–13347 20624969 10.1073/pnas.1002622107 PMC2922147

[22] Katzmann D. J. Odorizzi G. Emr S. D. (2002) Receptor downregulation and multivesicular-body sorting. Nat. Rev. Mol. Cell Biol. 3, 893–905 12461556 10.1038/nrm973

[23] Medina R. J. O'Neill C. L. Devine A. B. Gardiner T. A. Stitt A. W. (2008) The pleiotropic effects of simvastatin on retinal microvascular endothelium has important implications for ischaemic retinopathies. PLoS One. 3, e2584 18612412 10.1371/journal.pone.0002584 PMC2440506

[24] Simons K. Ikonen E. (1997) Functional rafts in cell membranes. Nature 387, 569–572 9177342 10.1038/42408

[25] Rothberg K. G. Heuser J. E. Donzell W. C. Ying Y. S. Glenney J. R. Anderson R. G. (1992) Caveolin, a protein component of caveolae membrane coats. Cell 68, 673–682 1739974 10.1016/0092-8674(92)90143-z

[26] Pelkmans L. Kartenbeck J. Helenius A. (2001) Caveolar endocytosis of simian virus 40 reveals a new two-step vesicular-transport pathway to the ER. Nature Cell Biol. 3, 473–483 11331875 10.1038/35074539

[27] Le P. U. Guay G. Altschuler Y. Nabi I. R. (2002) Caveolin-1 is a negative regulator of caveolae-mediated endocytosis to the endoplasmic reticulum. J. Biol. Chem. 277, 3371–3379 11724808 10.1074/jbc.M111240200

[28] Simons K. Toomre D. (2000) Lipid rafts and signal transduction. Nat. Rev. Mol. Cell Biol. 1, 31–39 11413487 10.1038/35036052

[29] Park J. H. Han H. J. (2009) Caveolin-1 plays important role in EGF-induced migration and proliferation of mouse embryonic stem cells: involvement of PI3K/Akt and ERK. Am. J. Physiol. Cell Physiol. 297, 935–944 10.1152/ajpcell.00121.2009 19625610

[30] Staubach S. Hanisch F. G. (2011) Lipid rafts: signaling and sorting platforms of cells and their roles in cancer. Expert Rev. Proteomics. 8, 263–277 21501018 10.1586/epr.11.2

[31] Fujioka Y. Tsuda M. Hattori T. Sasaki J. Sasaki T. Miyazaki T. Ohba Y. (2011) The Ras-PI3K signaling pathway is involved in clathrin-independent endocytosis and the internalization of influenza viruses. PLoS One 6, 16324 10.1371/journal.pone.0016324 PMC3024431 21283725

[32] Razandi M. Pedram A. Levin E. R. (2010) Heat shock protein 27 is required for sex steroid receptor trafficking to and functioning at the plasma membrane. Mol. Cell. Biol. 30, 3249–3261 20439495 10.1128/MCB.01354-09 PMC2897588

[33] Lavoie J. N. Hickey E. Weber L. A. Landry J. (1993) Modulation of actin microfilament dynamics and fluid phase pinocytosis by phosphorylation of heat shock protein 27. J. Biol. Chem. 268, 24210–24214 8226968

[34] Chen H. Zheng C. Zhang Y. Chang Y. Z. Qian Z. M. Shen X. (2006) Heat shock protein 27 downregulates the transferrin receptor 1-mediated iron uptake. Int. J Biochem. Cell Biol. 38, 1402–1416 16546437 10.1016/j.biocel.2006.02.006

[35] Sasai K. Kakumoto K. Hanafusa H. Akagi T. (2007) The Ras-MAPK pathway downregulates Caveolin-1 in rodent fibroblast but not in human fibroblasts: implications in the resistance to oncogene-mediated transformation. Oncogene 26, 449–455 16832346 10.1038/sj.onc.1209792

[36] Engelman J. A. Chu C. Lin A. Jo H. Ikezu T. Okamoto T. Kohtz D. S. Lisanti M. P. (1998) Caveolin-mediated regulation of signaling along the p42/44 MAP kinase cascade in vivo. A role for the caveolin-scaffolding domain. FEBS Lett. 428, 205–211 9654135 10.1016/s0014-5793(98)00470-0

[37] Gosens R. Stelmack G. L. Dueck G. McNeill K. D. Yamasaki A. Gerthoffer W. T. Unruh H. Gounni A. S. Zaagsma J. Halayko A. J. (2006) Role of caveolin-1 in p42/p44 MAP kinase activation and proliferation of human airway smooth muscle. Am. J. Physiol. Lung Cell Mol. Physiol. 291, L523–L534 16617096 10.1152/ajplung.00013.2006

[38] Williams T. Lisanti M. (2005) Caveolin-1 in oncogenic transformation, cancer, and metastasis. Am. J. Physiol. Cell Physiol. 288, C494–C506 15692148 10.1152/ajpcell.00458.2004

[39] McCubrey J. A. Steelman L. S. Chappell W. H. Abrams S. L. Wong E. W. Chang F. Lehmann B. Terrian D. M. Milella M. Tafuri A. Stivala F. Libra M. Basecke J. Evangelisti C. Martelli A. M. Franklin R. A. (2007) Roles of the Raf/MEK/ERK pathway in cell growth, malignant transformation and drug resistance. Biochim. Biophys. Acta. 1773, 1263–1284 17126425 10.1016/j.bbamcr.2006.10.001 PMC2696318

[40] Koppers-Lalic D. Hogenboom M. M. Middeldorp J. M. Pegtel D. M. (2013) Virus-modified exosomes for targeted RNA delivery; A new approach in nanomedicine. Adv. Drug Deliv. Rev. 65, 348–356 22820525 10.1016/j.addr.2012.07.006 PMC7103310

